# Influenza Vaccination and Incident Tuberculosis among Elderly Persons, Taiwan^1^

**DOI:** 10.3201/eid2403.152071

**Published:** 2018-03

**Authors:** Yung-Feng Yen, Sheng-Wei Pan, Vincent Yi-Fong Su, Pei-Hung Chuang, Jia-Yih Feng, Wei-Juin Su

**Affiliations:** National Yang-Ming University, Taipei, Taiwan (Y.-F. Yen, S.-W. Pan, V.Y.-F. Su, J.-Y. Feng, W.-J. Su);; Taipei City Hospital, Taipei (Y.-F. Yen, V.Y.-F. Su);; Taipei Veterans General Hospital, Taipei (S.-W. Pan, P.-H. Chuang, J.-Y. Feng, W.-J. Su)

**Keywords:** influenza vaccination, tuberculosis and other mycobacteria, Taiwan, elderly, influenza, viruses, bacteria, vaccines

## Abstract

Experimental studies have demonstrated that influenza vaccination may protect against tuberculosis (TB) through a Th17 response. This nationwide cohort study aimed to evaluate the association of influenza vaccination with incident TB among elderly persons in Taiwan. This 2005–2012 study included 99,982 elderly persons (64,290 vaccinated and 35,692 unvaccinated) from the Taiwan National Health Insurance Research Database. During the 738,367 person-years of follow-up, 1,141 (1.14%) persons had incident TB. The cumulative incidences of TB were 145.2 cases/100,000 person-years among vaccinated elderly persons and 175.5 cases/100,000 person-years among unvaccinated elderly persons (p = 0.002). The time-dependent Cox proportional hazards model revealed that influenza vaccination was an independent protective factor for incident TB. Our results suggest that influenza vaccination is associated with a lower risk of incident TB among elderly persons in Taiwan. Further investigation of biologic mechanisms is warranted.

Tuberculosis (TB) remains a common and deadly disease throughout the world (*1*). In 2014, there were 9.6 million cases of TB worldwide, and 1.5 million persons died of this disease (*2*). When *Mycobacterium tuberculosis* infects a host, T-cell–mediated immunity plays a major role in protecting against the development of TB. In this context, *M. tuberculosis*–induced T-cell–mediated immunity activates macrophage phagocytosis and kills intracellular *M. tuberculosis* (*3*). Activation of helper T cells also drives their secretion of tumor necrosis factor α, which attracts additional macrophages and lymphocytes and promotes granuloma formation to control the spread of *M. tuberculosis* (*3*). Previous studies have also demonstrated that the *M. bovis* bacillus Calmette-Guerin vaccine effectively protects infants from TB through the activation of conventional T-cell immunity (*4*,*5*). Although there is no effective vaccine to prevent TB in older adults, a recent report has demonstrated that a partially effective (e.g., 60% efficacy) vaccine targeted at adults can reduce the prevalence of TB (*6*).

Influenza vaccination is a safe and effective method of preventing influenza infections in elderly persons (*7**–**9*). A previous study demonstrated that the activation of CD4+ T cells (Th1) by influenza vaccination leads to the secretion of Th1-type cytokines (e.g., interferon-γ) (*10*), which activates macrophage phagocytosis and may subsequently kill intracellular *M. tuberculosis*. Recent animal studies have also demonstrated that influenza vaccination may provide protection against a range of pathogens (e.g., *M. tuberculosis*) by activating a Th17 response (*11*). Although several reports have indicated a possible interaction between influenza vaccination and TB, little is known regarding the association between influenza vaccine and subsequent TB development. Therefore, we conducted a nationwide, population-based cohort study during 2005–2012 to evaluate the association between the influenza vaccine and incident TB among elderly persons in Taiwan.

## Methods

### Background Information

The National Health Insurance (NHI) program in Taiwan is a universal and comprehensive program that was implemented on March 1, 1995 (*12*). The NHI program covers inpatient, outpatient, and emergency care, as well as alternative medicine, dental services, and prescription drugs. More than 99% of Taiwan’s population is enrolled in the NHI program (*12*). Since 2001, Taiwan’s government has implemented a free annual influenza vaccination program for elderly persons (>65 years of age) (*13*).

### Study Population

This nationwide cohort study used the National Health Insurance Research Database (NHIRD), which is managed by the Taiwan National Health Research Institutes. The Longitudinal Health Insurance Database, a subset of the NHIRD, is a representative database of 1 million persons randomly sampled by the National Health Research Institutes from the registry of all NHI enrollees. The NHIRD can be accessed at http://nhird.nhri.org.tw/en/index.htm, and the data are offered to scientists for research purposes. Personally identifiable information is encrypted. The NHIRD contains comprehensive medical information for insured persons, which includes drug prescriptions (e.g., influenza vaccinations) and diagnostic codes using the format of the International Classification of Diseases, Ninth Revision, Clinical Modification (ICD-9-CM). The accuracy of major disease diagnoses (e.g., diabetes mellitus and cerebrovascular disease [CVD]) in the NHIRD has been well validated (*14*,*15*).

We selected persons who were >65 years of age in 2005. Those who had received a TB diagnosis (ICD-9-CM codes 010–018) before enrollment were excluded. All persons were followed until a TB diagnosis, death, or December 31, 2013. This study was approved by the institutional review board of Taipei Veterans General Hospital (IRB 2015-04-004AC).

### Outcome Variable

The outcome variable was new-onset TB during the follow-up period. Incident TB was defined using the presence of the appropriate ICD-9-CM codes (010–018) (*16*) and the prescription of >2 anti-TB drugs (isoniazid, ethambutol, rifampin, or pyrazinamide) for 4 weeks.

### Main Explanatory Variable

The main explanatory variable was influenza vaccination, which was identified using the drug codes for influenza vaccination (*17*). The free annual influenza vaccination program for elderly persons in Taiwan usually begins at the beginning of October and continues through the end of April in the following year. The influenza season in Taiwan was typically defined as the period from October through the following March (*17*). The vaccines in the influenza immunization program were Fluvirin (Novartis Vaccines, Basel, Switzerland); Vaxigrip (Pasteur Merieux Connaught, Lyon, France); and Fluarix (GlaxoSmithKline, Research Triangle Park, NC, USA). The status of influenza vaccination was recorded in each influenza vaccination program for all elderly persons in Taiwan; time-dependent Cox proportional hazard models were used to evaluate the association between influenza vaccination and incident TB. For descriptive analysis, we defined 2 groups on the basis of whether or not they had ever received an influenza vaccination.

### Controlling Variables

The controlling variables in this study were the persons’ sociodemographic characteristics (income level and urbanization) and concurrent conditions. Income level was calculated using the average monthly income of the insured person; persons were categorized into 3 levels: low (<20,000 new Taiwan dollars [NTD]), intermediate (20,000 NTD to <40,000 NTD), or high (>40,000 NTD). Urbanization was categorized as whether the person lived in an urban, suburban, or rural area. Concurrent conditions were diabetes (ICD-9 code 250), congestive heart failure (CHF; ICD-9 code 428.0), CVD (ICD-9 codes 430–437, excluding 432), hypertension (ICD-9 codes 401–405), chronic kidney disease (CKD; ICD-9 codes 580–587), cancer (ICD-9 codes 140–208), chronic obstructive pulmonary disease (COPD; ICD-9 codes 491, 492, and 496), and asthma (ICD-9 code 493). A person was considered to have a concurrent condition only if the condition occurred in an inpatient setting or at >2 outpatient visits (*18*).

### Validation of TB Diagnosis

Identification of patients with an ICD-9-CM code for TB was validated by analysis of selected samples from the claims database of Taipei Veterans General Hospital (a 2,800-bed tertiary referral hospital in Taiwan) for 2014. The content of this database is used for reimbursement and is similar to that of the NHIRD. Two pulmonologists (V.Y.-F.S. and S.-W.P.) independently reviewed the clinical and laboratory data from the selected samples. We used a positive TB culture as the standard for the diagnosis of TB.

### Statistical Analysis

Continuous sociodemographic data were presented as mean (SD); we used the 2-sample *t*-test for intergroup comparisons. We analyzed categorical data using the Pearson χ^2^ test, as appropriate. We calculated the incidence of TB per 100,000 person-years for vaccinated and unvaccinated elderly persons and estimated the hazard ratios (HRs) of incident TB among vaccinated elderly persons (compared with unvaccinated elderly patients) using Cox proportional hazard models. We defined the exposed preventive fraction as the theoretically preventable proportion of the incidence rate of the outcome of interest (incident TB) in the exposed population if the influenza vaccination had been implemented for all persons. We calculated the preventive fraction using the equation (*19*) preventive fraction among exposed persons = [(CI_u_) – (CI_e_)] / (CI_u_) = 1 – relative risk, where CI_u_ is the cumulative incidence in the unexposed group (no influenza vaccination) and CI_e_ is the cumulative incidence in the exposed group (influenza vaccination).

We defined the population preventive fraction as the theoretically preventable proportion of the incidence rate of the outcome of interest (incident TB) in the entire population if the influenza vaccination had been implemented for all persons. We calculated the population preventive fraction using the equation population preventive fraction = (proportion of exposed cases) × (preventive fraction in the exposed cases), where the proportion of exposed cases is the prevalence of influenza vaccination.

We analyzed all data regarding influenza vaccination that were recorded in each influenza vaccination program for all persons. In addition, we analyzed the status of concurrent conditions as collected annually among elderly patients. Thus, information regarding the change in vaccination and status of concurrent conditions was available for each person throughout the study period. Therefore, we used Cox proportional hazards models with time-dependent covariates (*20*) to evaluate the associations of influenza vaccination with incident TB. In these models, influenza vaccination and concurrent conditions were considered the time-dependent variables (*20*), whereas potential confounders (e.g., age, sex, and income level) that were collected at baseline were considered fixed covariates. Because a previous report showed that influenza vaccine-induced immunity could last for 4 months in the elderly population (*21*), we hypothesized the duration of influenza vaccine-induced immunity as 4 months in the time-dependent Cox proportional hazards model. The formula for the Cox proportional hazards model is log *h_i_*(*t*) = log *h*_0_(*t*) + β_1_*x_i_*_1_ + β_2_*x_i_*_2_ + … + β*_k_x_ik_*) + [λ_1_*x_j_*_1_(*t*) + λ_2_*x_j_*_2_(*t*) + … λ*_k_x_jk_*(*t*)], where *x_i_*_1_ = (age, sex, and income level) are the fixed variables and *x_j_*_1_ = (influenza vaccination and concurrent conditions) are the time-dependent variables.

We examined the proportional hazard assumption by plotting the log–log plots according to influenza vaccination status; the plot showed lines for elderly persons in Taiwan with and without influenza vaccination that were straight and parallel. Adjusted HRs with 95% CIs were reported to indicate the strength and direction of the associations. To examine the interaction between influenza vaccination and other covariates in the multivariate analysis, we conducted subgroup analyses after stratifying study subjects by sex, age, and concurrent conditions. We conducted all data management and analyses using SAS software version 9.4 (SAS Institute, Cary, NC, USA).

## Results

### Patient Selection

This study identified 102,123 elderly persons in Taiwan who were >65 years of age in 2005. After excluding persons with antecedent TB (n = 568) and those with incomplete data (n = 1,573), we included the remaining 99,982 elderly persons in our analyses (Figure). During the follow-up period, 64.3% (64,290) of the elderly persons received influenza vaccination; 14.2% (14,167) were vaccinated once, 10.3% (10,277) twice, 8.6% (8,615) 3 times, 7.8% (7,800) 4 times, 6.9% (6,815) 5 times, 6.2% (6,188) 6 times, 5.6% (5,591) 7 times, and 4.8% (4,801) 8 times. The mean (SD) age was 73.7 (7.0) years and 49.5% were men. During the 738,367 person-years of follow-up, we observed 1,141 (1.14%) incident TB cases.

**Figure:**
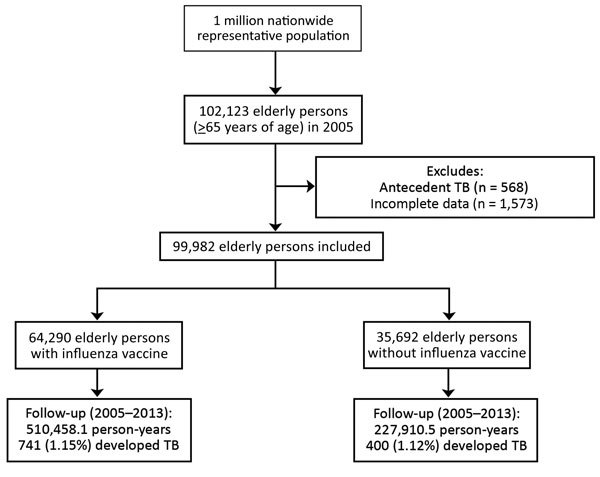
Flowchart showing enrollment and follow-up for elderly persons with and without influenza vaccination, Taiwan, 2005–2012. TB, tuberculosis.

### Demographic Characteristics and Concurrent Conditions among Elderly Persons

We compiled demographic characteristics and concurrent conditions among elderly persons in Taiwan with and without vaccination (Table 1). Elderly persons with vaccination were slightly younger than those without vaccination (73.2 vs. 74.6 years of age). In addition, compared with unvaccinated elderly persons, vaccinated elderly persons had a higher proportion of some concurrent conditions (e.g., diabetes, COPD, asthma, and hypertension) and a lower proportion of other concurrent conditions (e.g., CHF, CKD, and cancer). We found no significant difference in the proportion of incident TB cases between vaccinated and unvaccinated elderly persons (1.15% vs. 1.12%).

**Table 1:** Characteristics of elderly persons with and without influenza vaccination, Taiwan, 2005–2012*

Characteristic	With vaccination, n = 64,290	Without vaccination, n = 35,692	p value
Age, y, mean ± SD	73.2 ± 6.3	74.6 ± 8.2	<0.001
65–69	23,339 (36.3)	12,321 (34.5)	<0.001
70–74	17,848 (27.8)	7,842 (22.0)	
>75	23,103 (35.9)	15,529 (43.5)	
Sex			
F	32,426 (50.4)	18,042 (50.6)	0.734
M	31,864 (49.6)	17,650 (49.4)	
Income level			
Low	44,867 (69.8)	25,250 (70.7)	<0.001
Intermediate	11,353 (17.7)	5,692 (16.0)	
High	8,070 (12.5)	4,750 (13.3)	
Urbanization			
Urban	14,767 (23.0)	10,245 (28.7)	<0.001
Suburban	38,331 (59.6)	20,817 (58.3)	
Rural	11,192 (17.4)	4,630 (13.0)	
Concurrent conditions			
Diabetes	14,085 (21.9)	6,944 (19.5)	<0.001
CHF	3,200 (5.0)	2,257 (6.3)	<0.001
COPD	12,310 (19.2)	5,881 (16.5)	<0.001
Asthma	6,491 (10.1)	2,948 (8.3)	<0.001
CVD	10,824 (16.8)	5,938 (16.6)	0.419
HTN	36,444 (56.7)	16,998 (47.6)	<0.001
CKD	4,790 (7.5)	2,937 (8.2)	<0.001
Cancer	4,142 (6.4)	2,731 (7.7)	<0.001
Outcomes			
New-onset TB	741 (1.15)	400 (1.12)	0.649
Follow-up years, mean (SD)	7.9 (2.0)	6.4 (3.3)	<0.001
Total follow-up duration, person-years	510,458.1	227,910.5	<0.001

*Values are no. (%) persons unless otherwise indicated. CHF, congestive heart failure; CKD, chronic kidney disease; COPD, chronic obstructive pulmonary disease; CVD, cerebrovascular disease; HTN, hypertension; TB, tuberculosis.

### Incidence Rate of TB

During the follow-up period, new-onset TB was diagnosed in 1,141 elderly patients. The cumulative incidence of TB was 145.2 cases/100,000 person-years among vaccinated elderly patients and 175.5 cases/100,000 person-years among unvaccinated elderly patients (Table 2). The HR of incident TB was 0.83 (95% CI 0.73–0.93) between vaccinated and unvaccinated elderly patients. The exposed fraction for incident TB was 17.3%, and the population preventive fraction for incident TB was 11.1%.

**Table 2:** Univariate and multivariate analyses of the protective and risk factors for incident TB among elderly persons with and without influenza vaccination, Taiwan, 2005–2012*

Characteristic	Person-years	Incidence density†	Univariate analysis, HR (95% CI)	Multivariate analysis, adjusted HR (95% CI)
Influenza vaccination				
No	227,910.5	175.5	1	1
Yes	510,458.1	145.2	0.83 (0.73–0.93)‡	0.82 (0.73–0.93)‡
Age, y				
65–69	293,257.6	106.4	1	1
70–74	197,971.8	150.5	1.41 (1.21–1.66)§	1.18 (1.00–1.38)¶
>75	247,139.3	214.9	2.02 (1.75–2.32)§	1.43 (1.24–1.66)§
Sex				
F	385,893.5	62.7	1	1
M	352,475.2	255.1	4.07 (3.53–4.69)§	3.32 (2.88–3.84)§
Income level				
Low	507,842.5	170.9	1	1
Intermediate	133,417.7	122.9	0.72 (0.61–0.85)§	0.88 (0.74–1.04)
High	97,108.5	112.2	0.66 (0.54–0.80)§	0.82 (0.67–1.00)
Urbanization				
Urban	187049.0	129.4	1	1
Suburban	436275.1	157.9	1.22 (1.05–1.41)‡	1.13 (0.98–1.31)
Rural	115044.5	182.5	1.41 (1.17–1.70)§	1.37 (1.13–1.65)¶
Concurrent conditions				
Diabetes				
No	596,098.4	153.5	1	1
Yes	142,270.2	158.9	1.03 (0.89–1.20)	1.17 (1.02–1.33)¶
CHF				
No	708,633.3	152.4	1	1
Yes	29,735.4	205.1	1.34 (1.04–1.74)¶	1.37 (1.16–1.62)§
COPD				
No	621,742.5	133.3	1	1
Yes	116,626.2	267.5	2.00 (1.76–2.28)§	2.77 (2.43–3.15)§
Asthma				
No	676,539.1	145.9	1	1
Yes	61,829.5	249.1	1.71 (1.44–2.02)§	1.12 (0.96–1.30)
CVD				
No	631659.3	152.0	1	1
Yes	106709.3	169.6	1.11 (0.95–1.31)	0.89 (0.78–1.02)
HTN				
No	360268.3	156.5	1	1
Yes	378100.3	152.6	0.97 (0.87–1.09)	0.86 (0.76–0.98)¶
CKD				
No	693223.9	149.2	1	1
Yes	45144.7	237.0	1.59 (1.30–1.94)§	1.31 (1.12–1.53)§
Cancer				
No	696681.8	150.9	1	1
Yes	41686.8	215.9	1.43 (1.15–1.77)‡	1.96 (1.71–2.26)§

*CHF, congestive heart failure; CKD, chronic kidney disease; COPD, chronic obstructive pulmonary disease; CVD, cerebrovascular disease; HR, hazard ratio; HTN, hypertension; TB, tuberculosis. †Per 100,000 person-years. ‡p<0.01. §p<0.001. ¶p<0.05.

### Protective and Risk Factors for Incident TB

We used a time-dependent Cox proportional hazards model to identify the independent protective and risk factors for incident TB (Table 2). After adjusting for the sociodemographic characteristics and concurrent conditions, we found that the risk of incident TB was 18% lower (95% CI 0.73–0.93) among elderly persons with influenza vaccination compared with unvaccinated persons. Another protective factor for incident TB was HTN. The risk factors for incident TB were age 70–74 years or >75 years, male sex, living in a rural area, diabetes, CHF, COPD, CKD, and cancer.

### Sensitivity Analyses

We conducted sensitivity analyses regarding the associations of influenza vaccination with incident TB and stratified results according to age group, sex, and concurrent conditions (Table 3). Time-dependent Cox regression analyses revealed that influenza vaccination significantly reduced the risk of incident TB in men; persons with low or high income; persons living in urban or suburban areas; persons with COPD or CVD; and persons without diabetes, CHF, asthma, HTN, CVD, cancer, or CKD.

**Table 3:** Sensitivity analysis for the associations of influenza vaccination with incident TB after adjusting for demographic and medical characteristics, Taiwan, 2005–2012*

Study subgroup	Incident TB, adjusted HR (95% CI)
All persons, n = 99,982	0.82 (0.73–0.93)‡
Age 65–69 y, n = 35,660	0.81 (0.64–1.03)
Age >70 y, n = 64,322	0.85 (0.67–1.08)
Male patients, n = 49,514	0.81 (0.70–0.93)‡
Female patients, n = 50,468	0.89 (0.68–1.17)
Patients with low income, n = 70,117	0.82 (0.71–0.94)‡
Patients with intermediate income, n = 17,045	1.04 (0.75–1.45)
Patients with high income, n = 12,820	0.62 (0.40–0.97)†
Patients in urban area, n = 25,012	0.71 (0.53–0.95)†
Patients in suburban area, n = 59,148	0.82 (0.70–0.96)†
Patients in rural area, n = 15,822	0.97 (0.73–1.28)
Diabetes patients, n = 21,029	0.80 (0.61–1.06)
Patients without diabetes, n = 78,953	0.83 (0.72–0.95)‡
Patients with CHF, n = 5,457	0.75 (0.43–1.30)
Patients without CHF, n = 94,525	0.82 (0.72–0.93)‡
Patients with COPD, n = 18,191	0.74 (0.58–0.94)†
Patients without COPD, n = 81,791	0.88 (0.76–1.02)
Patients with asthma, n = 9,439	0.79 (0.57–1.10)
Patients without asthma, n = 90,543	0.83 (0.73–0.95)‡
Patients with HTN, n = 53,442	0.86 (0.72–1.02)
Patients without HTN, n = 46,540	0.80 (0.66–0.96)†
Patients with CVD, n = 16,762	0.70 (0.51–0.97)†
Patients without CVD, n = 83,220	0.85 (0.74–0.97)†
Patients with cancer, n = 6,873	0.85 (0.55–1.31)
Patients without cancer, n = 93,109	0.81 (0.71–0.92)‡
Patients with CKD, n = 7,727	0.80 (0.53–1.20)
Patients without CKD, n = 92,255	0.82 (0.72–0.94)‡

*CHF, congestive heart failure; CKD, chronic kidney disease; COPD, chronic obstructive pulmonary disease; CVD, cerebral vascular disease; HR, hazard ratio; HTN, hypertension; TB, tuberculosis. †p<0.05. ‡p<0.01.

### Dose-Response Relationship between Influenza Vaccination and Incident TB

We evaluated the dose-response relationship between influenza vaccination and incident TB using the Cox proportional hazards models. The risk of developing TB (adjusted HR 0.81; 95% CI 0.79–0.83) decreased as the number of influenza vaccinations increased.

### Validation

During January 1, 2014–December 31, 2014, a total of 433 patients had an ICD-9-CM code for TB. Of these patients, 326 had prescriptions for >2 anti-TB drugs for 4 weeks and were selected for validation. Among these 326 patients, the diagnosis of TB was confirmed in 314 and excluded in 12 (specificity 96.3%).

## Discussion

Our longitudinal study aimed to evaluate the temporal association of influenza vaccination with incident TB during the 9-year follow-up period. The results indicate that, after adjusting for demographic data, concurrent conditions, and income level, elderly persons in Taiwan with influenza vaccination had a lower risk of incident TB than unvaccinated elderly persons. Our study also conducted a sensitivity analysis of influenza vaccination with incident TB after stratifying persons according to age, sex, and concurrent conditions. Influenza vaccination significantly reduced the risk of incident TB in men; persons with low or high income; persons living in urban or suburban area; persons with COPD or CVD; and persons without diabetes, CHF, asthma, HTN, CVD, cancer, or CKD.

Activation of T-cell immunity by influenza vaccination may contribute to our observed lower risk of incident TB among vaccinated elderly Taiwanese patients. Activation of CD4+ Th1-type cells by influenza vaccination induces the secretion of Th1-type cytokines (e.g., interferon-γ) (*10*), which can activate macrophage phagocytosis and kill intracellular *M. tuberculosis*. The activation of Th1-type cells by influenza vaccination also accelerates granuloma formation and controls the spread of MTB (*3*). Furthermore, recent animal studies have demonstrated that influenza vaccination may provide protection against TB by initiating a Th17 response, which causes the recruitment of neutrophils, release of antimicrobial peptides, and interleukin-17–driven Th1 immunity (*11*). All these mechanisms indicate that the activation of helper T-cell immunity by influenza vaccination may contribute to the reduced risk of incident TB among vaccinated elderly persons.

Our study also revealed that the population preventive fraction of influenza vaccination for incident TB was 11.1%. Although influenza vaccination reduced the risk of incident TB by only 18% in an older population, a previous report showed that a relevant vaccine (when given to adults) would reduce the burden of TB, even when the vaccine is only partially effective in protecting against *M. tuberculosis* infection (*6*). Therefore, our findings suggest that influenza vaccination should be offered to elderly persons to prevent infections.

The aim of this cohort study was to determine the association between influenza vaccination and subsequent TB development. Our research design, which included unbiased patient selection and strict TB diagnostic criteria, supports the validity of these findings. In Taiwan, TB has been the most prevalent notified infectious disease for decades (*22*), and TB-related data are strengthened by the tight regulation of the TB reporting system in Taiwan. By law, clinics and hospitals in Taiwan must report TB cases to Taiwan’s Centers for Disease Control within 7 days (*22*). Moreover, Taiwan’s Centers for Disease Control convenes monthly expert committee meetings to discuss ambiguous reported TB cases (*22*). Our study also examined the internal validity of TB diagnosis classification using the same ICD-9-CM coding and confirmed that interobserver agreement and accuracy in identifying TB cases were excellent.

The lower risk of incident TB among vaccinated elderly patients is likely related to activation of T-cell–mediated immunity. A particular strength of this study is that we traced all elderly persons with minimal referral bias, because all medical care is covered by the Taiwan NHI program. Furthermore, the study’s large sample size was sufficiently powered to detect the real, albeit subtle, difference between vaccinated and unvaccinated elderly persons. In addition, the annual influenza vaccination status for all persons was collected, and influenza vaccination was considered a time-dependent variable in the multivariate analysis. Longitudinal studies that do not account for changes in the annual vaccination will not be able to produce precise estimates of the vaccination’s effect on incident TB (*23*).

This study has several limitations. First, some potential risk factors (e.g., smoking and obesity) were not available for our analysis. However, some smoking-related health effects would be partially reflected in the presentation of concurrent conditions (e.g., COPD and hypertension), which were included in our analysis. Second, influenza vaccination among elderly persons in Taiwan was voluntary, rather than randomly assigned. Therefore, unmeasured confounders (such as concurrent conditions) may exist for the association of influenza vaccination with incident TB and death from all causes. However, a previous study demonstrated that elderly persons in Taiwan with influenza vaccination have a greater number of concurrent conditions compared with their unvaccinated counterparts (*13*). Therefore, because persons with more chronic diseases have a higher risk of incident TB (*24*,*25*), this difference would lead to our underestimating the protective effect of influenza vaccination on TB development. Third, TB diagnoses that rely on administrative claims data (which are recorded by physicians or hospitals) may be less accurate than diagnoses that are made in a prospective clinical setting. Furthermore, the history of TB disease among elderly patients in Taiwan can be traced back only to 1995. However, there is no reason to suspect that the validity of claims data would differ with a patient’s TB status. Moreover, diagnosis of TB by ICD-9-CM coding was validated in this study, indicating that the accuracy is excellent. Finally, because the Taiwan government provides free influenza vaccination only for persons who are >65 years of age (*13*), we could evaluate the association between influenza vaccination and incident TB only among elderly persons. Therefore, the generalizability of our findings to younger persons requires further verification.

In conclusion, this nationwide long-term cohort study determined the associations of influenza vaccination with incident TB among elderly persons in Taiwan. Influenza vaccination was associated with lower risks of incident TB, after adjusting for demographic data, concurrent conditions, and income level. Nevertheless, more comprehensive studies are needed to confirm our findings and identify the potential biologic mechanism(s) that explain these associations.
